# Caspase-8 activation by cigarette smoke induces pro-inflammatory cell death of human macrophages exposed to lipopolysaccharide

**DOI:** 10.1038/s41419-023-06318-6

**Published:** 2023-11-25

**Authors:** Marta Cristaldi, Marco Buscetta, Maura Cimino, Agnese La Mensa, Maria Rita Giuffrè, Luigi Fiore, Claudia Carcione, Fabio Bucchieri, Francesca Rappa, Claudia Coronnello, Nicolina Sciaraffa, Santina Amato, Tommaso Silvano Aronica, Giovanna Lo Iacono, Alessandro Bertani, Elisabetta Pace, Chiara Cipollina

**Affiliations:** 1 Fondazione Ri.MED, Via Bandiera 11, 90133 Palermo, Italy; 2 Dipartimento di Biomedicina, Neuroscienze e Diagnostica Avanzata, Università di Palermo, Via del Vespro 129, 90127 Palermo, Italy; 3 Dipartimento di Scienze Biomediche, Odontoiatriche e delle Immagini Morfologiche e Funzionali, Università di Messina, Piazza Pugliatti, 1, 98122 Messina, Italy; 4 Istituto di Farmacologia Traslazionale (IFT)-CNR, Via Ugo la Malfa 153, 90146 Palermo, Italy; 5 Azienda di Rilievo Nazionale ed Alta Specializzazione Ospedali (A.R.N.A.S) “Civico Di Cristina Benfratelli”, Piazza Nicola Leotta 4, 90127 Palermo, Italy; 6 IRCCS ISMETT—UPMC Italy, Via E. Tricomi 1, 90127 Palermo, Italy

**Keywords:** Cell death and immune response, Innate immunity

## Abstract

Cigarette smoking impairs the lung innate immune response making smokers more susceptible to infections and severe symptoms. Dysregulation of cell death is emerging as a key player in chronic inflammatory conditions. We have recently reported that short exposure of human monocyte-derived macrophages (hMDMs) to cigarette smoke extract (CSE) altered the TLR4-dependent response to lipopolysaccharide (LPS). CSE caused inhibition of the MyD88-dependent inflammatory response and activation of TRIF/caspase-8/caspase-1 pathway leading to Gasdermin D (GSDMD) cleavage and increased cell permeability. Herein, we tested the hypothesis that activation of caspase-8 by CSE increased pro-inflammatory cell death of LPS-stimulated macrophages. To this purpose, we measured apoptotic and pyroptotic markers as well as the expression/release of pro-inflammatory mediators in hMDMs exposed to LPS and CSE, alone or in combination, for 6 and 24 h. We show that LPS/CSE-treated hMDMs, but not cells treated with CSE or LPS alone, underwent lytic cell death (LDH release) and displayed apoptotic features (activation of caspase-8 and -3/7, nuclear condensation, and mitochondrial membrane depolarization). Moreover, the negative regulator of caspase-8, coded by CFLAR gene, was downregulated by CSE. Activation of caspase-3 led to Gasdermin E (GSDME) cleavage. Notably, lytic cell death caused the release of the damage-associated molecular patterns (DAMPs) heat shock protein-60 (HSP60) and S100A8/A9. This was accompanied by an impaired inflammatory response resulting in inhibited and delayed release of IL6 and TNF. Of note, increased cleaved caspase-3, higher levels of GSDME and altered expression of cell death-associated genes were found in alveolar macrophages of smoker subjects compared to non-smoking controls. Overall, our findings show that CSE sensitizes human macrophages to cell death by promoting pyroptotic and apoptotic pathways upon encountering LPS. We propose that while the delayed inflammatory response may result in ineffective defenses against infections, the observed cell death associated with DAMP release may contribute to establish chronic inflammation.

**Figure:**
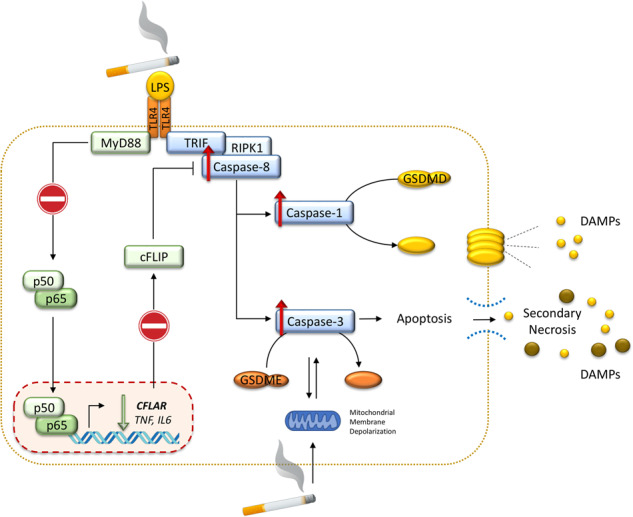
CS exposure sensitizes human macrophages to pro-inflammatory cell death. Upon exposure to LPS, CS inhibits the TLR4/MyD88 inflammatory response, downregulating the pro-inflammatory genes *TNF* and *IL6* and the anti-apoptotic gene *CFLAR*, known to counteract caspase-8 activity. CS enhances caspase-8 activation through TLR4/TRIF, with a partial involvement of RIPK1, resulting on the activation of caspase-1/GSDMD axis leading to increased cell permeability and DAMP release through gasdermin pores [19]. At later timepoints caspase-3 becomes strongly activated by caspase-8 triggering apoptotic events which are associated with mitochondrial membrane depolarization, gasdermin E cleavage and secondary necrosis with consequent massive DAMP release.

CS exposure sensitizes human macrophages to pro-inflammatory cell death. Upon exposure to LPS, CS inhibits the TLR4/MyD88 inflammatory response, downregulating the pro-inflammatory genes *TNF* and *IL6* and the anti-apoptotic gene *CFLAR*, known to counteract caspase-8 activity. CS enhances caspase-8 activation through TLR4/TRIF, with a partial involvement of RIPK1, resulting on the activation of caspase-1/GSDMD axis leading to increased cell permeability and DAMP release through gasdermin pores [19]. At later timepoints caspase-3 becomes strongly activated by caspase-8 triggering apoptotic events which are associated with mitochondrial membrane depolarization, gasdermin E cleavage and secondary necrosis with consequent massive DAMP release.

## Introduction

The detrimental impact of cigarette smoking on human health is widely recognized, but despite this, tobacco smoke remains a major cause of death worldwide [1, 2]. Cigarette smoke (CS) contributes to chronic inflammation underlying many airways diseases such as asthma [3], chronic obstructive pulmonary disease (COPD) [4, 5], and pulmonary fibrosis [6]. It is widely known that CS compromises the innate immune responses. Alveolar macrophages (AMs) from smoking subjects often display a totally altered immune phenotype, with defective expression of cytokines and recognition receptors, impaired phagocytosis/efferocytosis and ability of tissue repair; this affects the process of pathogen recognition/clearance and the inflammation resolution [1, 7–11]. As a result, smokers are more susceptible to bacterial/viral infections and develop more severe symptoms that easily become chronic. The correlation between cigarette smoking and the observed increased risk of infection worsening symptoms deserves a deeper investigation. Understanding CS-induced molecular alterations that hamper lung macrophages’ functions and deregulate inflammatory responses to pathogens may unveil new mechanisms to target for the development of new therapeutics.

Cell death is considered a double-edge sword: it has a physiological role on the regulation of tissue homeostasis and repair after injury and contributes to inflammation if out of control [12]. Increasing number of studies suggest that lytic forms of cell death such as pyroptosis, necroptosis, and NETosis, while contributing to block infections, under specific circumstances may establish a vicious cycle sustaining chronic inflammation [12]. Caspase-8 is central in most regulated cell death pathways and is considered both inflammatory and apoptotic. Caspase-8 mediates apoptosis by activating the effector caspase-3. Very recently, it has been reported that caspase-3 is able to cleave and activate Gasdermin E (GSDME) and this may turn apoptosis into secondary pyroptosis [13–15]. In addition, it has been reported that caspase-8 can promote pyroptosis by activating Gasdermin D (GSDMD), either by direct cleavage after Asp276 [13], or indirectly via caspase-1 activation [7, 16]. Due to its critical role in the control of cell death and survival, caspase-8 is regulated at multiple levels. The cell is equipped with several inhibitors that retain caspase-8 from being activated. Among these, the anti-apoptotic protein Cellular FLICE-like inhibitory protein (cFLIP) is a major negative regulator of caspase-8. cFLIP suppresses caspase-8 activation, therefore preventing complex II formation and downstream cell death pathways [17, 18].

In recent studies, we reported that cigarette smoke extract (CSE) alters the Toll-Like Receptor 4 (TLR4) dependent macrophage response to lipopolysaccharide (LPS). CSE inhibits the signaling dependent from Myeloid differentiation primary response 88 (MyD88), while activating caspase-1 and GSDMD via TIR-domain-containing adapter-inducing interferon-β (TRIF)-caspase-8 pathway independently from ASC-dependent inflammasomes. We showed that short exposure of human macrophages to CSE caused the formation of GSDMD pores on cell membrane, increasing macrophage permeability with no effect on cell viability [7, 19].

Herein, we further extended our investigation by exploring the impact of caspase-8 activation by CSE in macrophages exposed to bacterial LPS at longer timepoints. We analyzed the activation of cell death pathways with a focus on apoptosis and pyroptosis. Furthermore, the impact of CSE on the expression and release of pro-inflammatory cytokines, and release of damage-associated molecular patterns (DAMPs) following cell death was evaluated. In vitro key findings were also tested in a small set of lung tissue samples from smokers and non-smoking controls. In addition, two publicly available microarray datasets of smokers and non-smoking subjects [11, 20] have been used to further investigate the smokers/non-smokers differentially expressed genes.

## Results

### hMDMs exposed to CSE undergo lytic cell death after stimulation with LPS

**Fig. 1:**
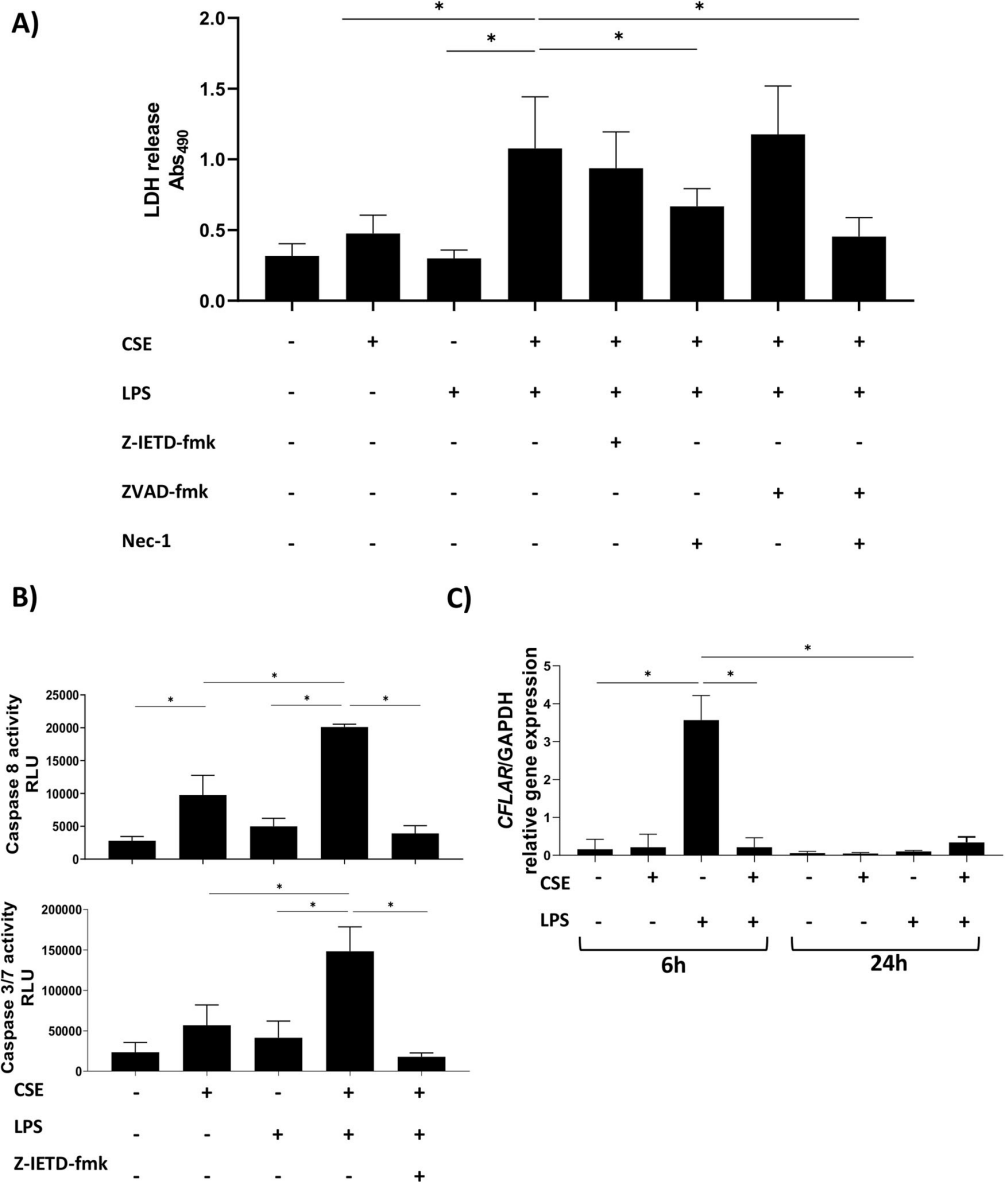
Lytic cell death occurred in hMDMs stimulated with LPS and CSE and was associated with enhanced caspase-8 and -3/7 activation and *CFLAR* inhibition. hMDMs were treated with 1 μg/ml of LPS and 20% CSE, alone or in combination. **A** LDH release and (**B**) extracellular activity of Caspase-8 and -3/7 (expressed as relative luminescence unit, RLU), were measured after 24 h stimulation. **C** *CFLAR* gene expression was measured after 6 and 24 h stimulation. Where indicated, cells were pre-treated for 1 h with 0.1 μM Z-IETD-fmk Caspase-8 inhibitor, 20 μM Z-VAD-fmk pan-caspase inhibitor, 50 μM Nec-1 RIPK1 inhibitor. The absorbance of LDH was measured at 490 nm. Data are presented as mean ± SEM (*N* = 3 independent donors).

To investigate the impact of CSE on cell fate of LPS-treated hMDMs, the occurrence of lytic cell death (measured as lactate dehydrogenase, LDH, release) was evaluated after co-stimulation with LPS and CSE, in combination or not, for 24 h. The involvement of caspases was evaluated by pre-treating the cells with Z-IETD caspase-8 inhibitor or Z-VAD pan-caspase inhibitor. As shown in Fig. 1A, LPS or CSE alone did not induce LDH release. On the contrary, lytic cell death occurred in hMDMs stimulated with LPS/CSE. LDH release was not inhibited by pre-treatment with Z-IETD or Z-VAD alone. It is known that under certain conditions, when caspase-8 activation is blocked, a “back-up” mechanism of cell death known as necroptosis is initiated by RIPK1 [13, 14]. In our model, the failure of Z-VAD and Z-IETD to reduce cell death was most probably due to the activation of necroptosis. Accordingly, treatment with Z-VAD in combination with the RIPK1 inhibitor Necrostatin-1 (Nec-1), significantly reduced the LDH release thus rescuing cells from death (Fig. 1A). Treatment with Nec-1 alone partially inhibited LPS/CSE-induced LDH release (Fig. 1A) as well as caspase-8 activation (Fig. S1).

### CSE enhances the activation of caspase-8 and -3/7 in response to LPS and downregulates *CFLAR* gene expression

To evaluate the involvement of caspase-8 and -3/7 in LPS/CSE-induced cell death, we analyzed the activity of caspase-8 and -3/7 in hMDMs exposed to LPS and CSE alone or in combination. CSE alone induced activation of caspase-8 that was accompanied with a mild activation of caspase-3/7. LPS alone did not induce caspase activation. Co-stimulation with LPS and CSE significantly increased the enzymatic activity of both caspase-8 and caspase-3/7 (Fig. 1B) compared to LPS or CSE alone. The activity of caspase-3/7 decreased following pre-treatment with the caspase-8 inhibitor Z-IETD (Fig. 1B). It is known that the activity of caspase-8 is strictly regulated by cFLIP, a major anti-apoptotic protein coded by *CFLAR* and induced by LPS stimulation [14, 17, 18]. RT-qPCR analysis (Fig. 1C) revealed that *CFLAR* gene expression increased 6 h after LPS stimulation, declining at 24 h. Of note, CSE completely blocked LPS-induced *CFLAR* gene expression (Fig. 1C).

### hMDMs exposed to LPS/CSE display apoptotic hallmarks

During apoptosis, nuclear chromatin becomes highly condensed; this process is followed by DNA fragmentation and packaging into apoptotic bodies [21, 22]. Likewise, mitochondria membrane depolarization and loss of the electrochemical gradient are specific markers of apoptosis execution [22–24]. Therefore, to investigate whether the observed activation of caspase-8 and -3/7 promoted progression into apoptotic cell death, we evaluated nuclear DNA condensation and mitochondrial membrane potential (MMP).

**Fig. 2:**
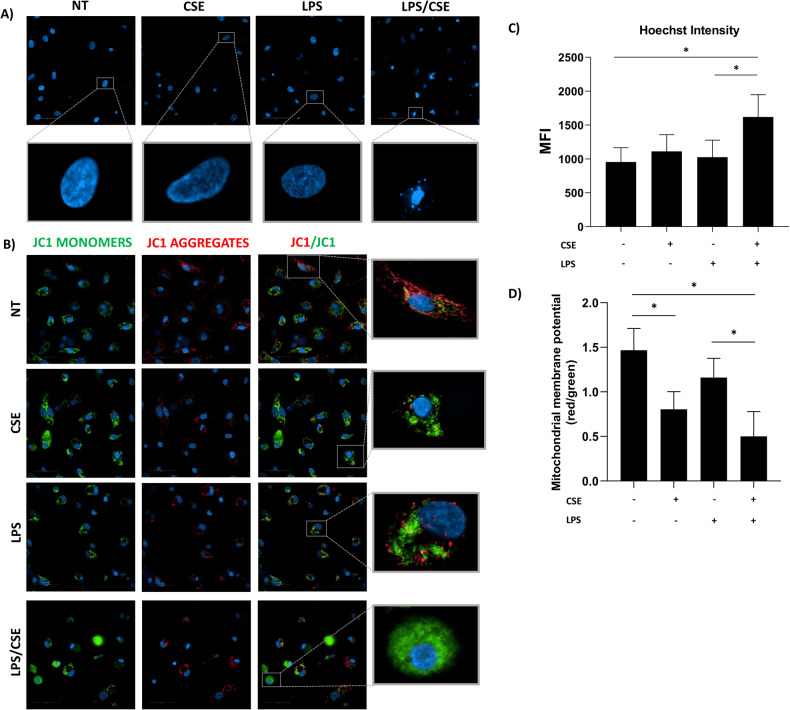
hMDMs exposed to CSE and LPS display apoptotic hallmarks. Representative images of hMDMs treated with 1 μg/ml of LPS and 20% CSE, alone or in combination, for 24 h and stained with (**A**) Hoechst 33342 for nuclear morphology evaluation and (**B**) JC-1 dye for MMP assessment. **C** Quantification of Hoechst 33342 intensity expressed as mean fluorescent intensity (MFI). **D** Quantification of the red (485/590 nm)/green (485/529 nm) fluorescence intensity ratio after JC1 staining. Scale bar: 50 μm. Data are presented as mean ± SEM (*N* = 3 independent donors).

Representative fluorescence images reported in Fig. 2A show increased nuclear dye intensity (indicative of nuclear condensation) and nuclear fragmentation in cells treated with LPS/CSE compared to all other conditions. Fluorescence quantification confirmed intensity increase in cells stimulated with LPS/CSE (Fig. 2C). MMP was evaluated after staining the cells with the JC1 probe. Representative confocal images of hMDMs treated with LPS/CSE, alone or in combination (Fig. 2B) show that all treatments caused a color shift of JC1 dye from red aggregates in non-treated hMDMs to green, indicative of electrochemical gradient loss and mitochondria membrane depolarization. The effect appeared stronger in cells exposed to CSE alone and in combination with LPS compared to the other conditions. The quantification of the ratio between red and green fluorescence confirmed the data derived from confocal microscopy analysis showing that both CSE alone and LPS/CSE caused depolarization of mitochondrial membrane (Fig. 2D).

### CSE induces caspase-8-dependent cleavage of GSDME in hMDMs exposed to LPS

**Fig. 3:**
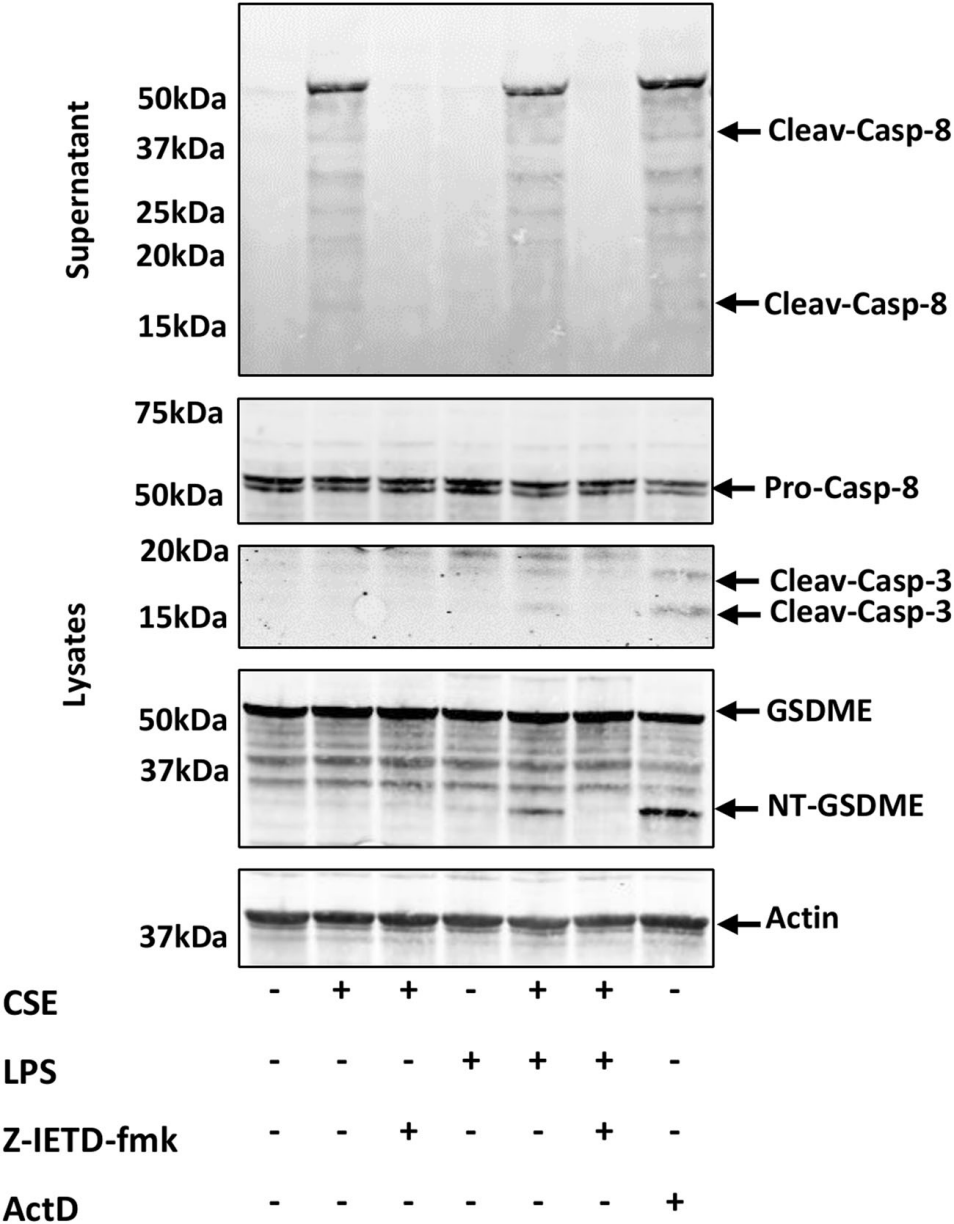
LPS triggers the activation of the axis caspase-8/caspase-3/GSDME in hMDMs exposed to CSE. Representative western blot images of Caspase-8, -3 and GSDME in hMDMs treated with 1 μg/ml of LPS and 20% CSE, alone or in combination for 24 h. Where indicated, hMDMs were pre-treated for 1 h with 0.1 μM Z-IETD-fmk Caspase-8 inhibitor (*N* = 3 independent donors). ActD 0.5 μg/ml was used as positive control. β-actin was used as loading control.

GSDME can be cleaved by caspase-3 after Asp270 to generate the GSDME-NT fragment that under specific circumstances may enhance cell death [25–27]. Considering the increased activity of caspase-8 and -3/7, and the increased LDH release from LPS/CSE-stimulated hMDMs, activation of GSDME was evaluated by western blot analysis. Treatment with the apoptosis inducer actinomycin D (ActD) was used as a positive control. In the same experiment, processing of caspase-8 and caspase-3 was evaluated. Treatment with CSE alone resulted in caspase-8 cleavage, consistent with the observed increase of enzymatic activity. However, no cleavage of caspase-3 and GSDME was observed in this condition. When cells exposed to CSE were stimulated with LPS, cleavage of caspase-8, caspase-3 and GSDME was observed (Fig. 3). Pre-treatment with the caspase-8 inhibitor Z-IETD blocked the cleavage of caspase-8, caspase-3 and GSDME (Fig. 3). To better assess the role of GSDME in LPS/CSE-induced cell death and caspase-3 activation, we silenced GSDME expression by a siRNA approach (Fig. S2A, B) and treated hMDMs with LPS and CSE, alone or in combination. LDH release was monitored over time (Fig. S2C). The release of the small DAMP S100A8/S100A9 was also monitored over time as alternative readout of pore formation and membrane rupture. Caspase-3 activation was assessed at 24 h (Fig. S3). We found that GSDME silencing did not reduce LPS/CSE-induced cell death, nor S100A8/A9 release or caspase-3 activation (Figs. S2 and S3).

### Increased cleaved caspase-3 and higher *GSDME* gene expression were observed in alveolar macrophages from smokers

**Fig. 4:**
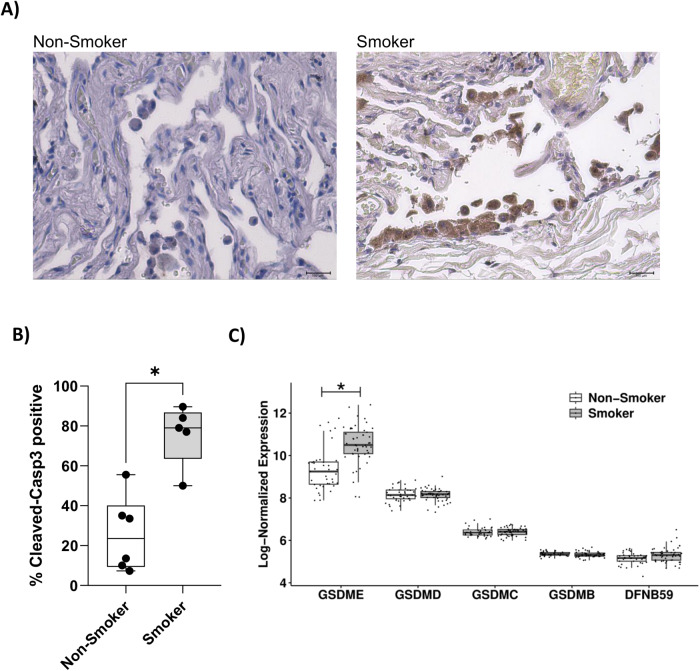
Cleaved Caspase-3 and *GSDME* gene expression in alveolar macrophages from smokers compared to non-smoking controls. **A** Representative images of immunohistochemical staining of distal lung tissue sections of Smokers (*N* = 5) and Non-smoking controls (*N* = 6), using a specific antibody for cleaved Caspase-3. **B** Graph showing the percentage of cells positive for cleaved Caspase-3. Data are presented as median with interquartile range. The difference between the percentage of immunopositivity in Non-Smoking controls and Smokers was statistically evaluated with the Mann–Whitney test. **C** Box plot of log-normalized expression of GSDM family genes in Non-Smokers and Smokers samples. Data are presented as median with interquartile range. Differences of Smokers vs. Non-Smokers gene expression were evaluated in the whole microarray with the moderated *t*-test and FDR correction.

We next evaluated whether cigarette smoking may promote the activation of caspase-8/caspase-3 pathway in human lung macrophages. To this purpose, activation of caspase-3 was evaluated by immunohistochemistry, using an antibody specifically recognizing cleaved caspase-3, in lung tissue sections of smoking subjects (*N* = 5). Results were compared with those obtained in non-smoking controls (*N* = 6). As shown in Fig. 4, the percentage of macrophages positively stained for cleaved caspase-3 was significantly higher in Smokers compared to Non-Smoker controls. To further investigate the activation of the key pathways identified in vitro, we also analyzed two publicly available gene expression datasets, where the gene expression profile of alveolar macrophages was evaluated in smokers and non-smoking controls. GSE13896 [11] and GSE2125 [20] datasets were merged to identify genes differentially expressed in Smokers (*N* = 49) compared to Non-Smokers (*N* = 39), on which a gene ontology (GO) enrichment analysis was performed with a focus on cell death terms. Figure 4C shows a significant increase of *GSDME* gene expression in Smoker patients compared to Non-Smokers. Moreover, among the GSDM family members the GSDME resulted the most highly expressed. Gene ontology enrichment analysis performed on differentially expressed genes revealed an over-representation of terms related to cell death processes (Fig. S4).

### Exposure to CSE delays the inflammatory response of hMDMs to LPS

**Fig. 5:**
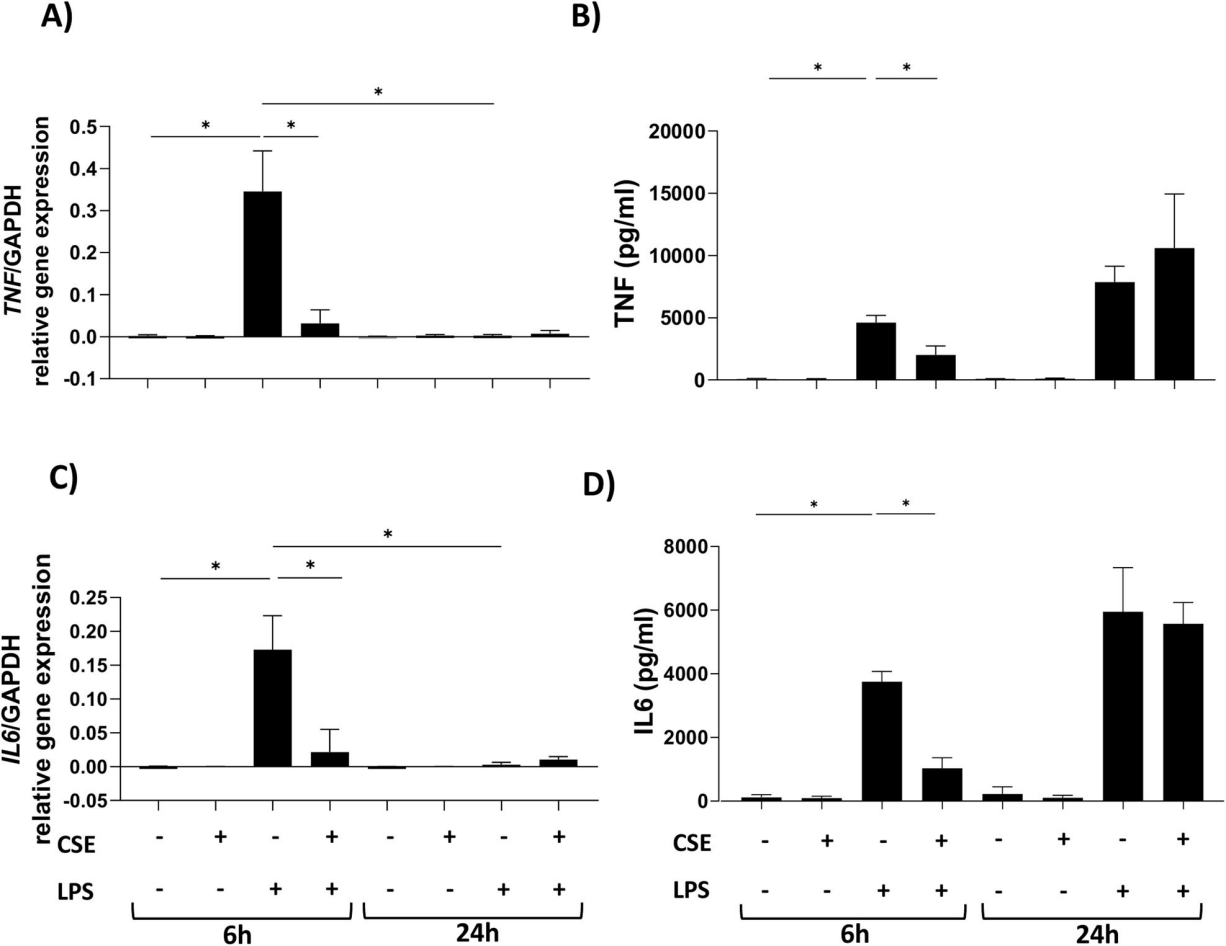
Exposure to CSE delayed the inflammatory response to LPS. Gene expression (left) and cytokine release (right) of (**A**, **B**) TNF and (**C**, **D**) IL6 from hMDMs exposed to 1 μg/ml of LPS and 20% CSE, alone or in combination, for 6 and 24 h. Data are presented as mean ± SEM (*N* = 3 independent donors).

Upon encountering pathogens, macrophages become quickly activated and release de novo produced pro-inflammatory mediators [25, 26]. Bacterial LPS activates an early transcriptional pro-inflammatory program right after binding to TLR4 and inducing the release of several pro-inflammatory cytokines, including TNF and IL6 [27]. To evaluate the impact of CSE on LPS-dependent inflammatory response, we assessed TNF and IL6 gene expression and cytokine release in hMDMs stimulated with LPS and CSE, alone or in combination, for 6 and 24 h. As expected, LPS induced a strong increase of *TNF* and *IL6* gene expression already at 6 h (Fig. 5A, C). This was accompanied by TNF and IL6 release (Fig. 5B, D). Gene expression declined at 24 h. CSE alone did not induce expression nor release of TNF and IL6 and inhibited LPS-induced transcriptional activation of TNF and IL6 (Fig. 5A, C). Consistently, LPS-induced protein release was strongly inhibited by CSE at 6 h. Interestingly, after 24 h stimulation no differences between LPS and LPS/CSE-treated cells were observed in the release of TNF and IL6 (Fig. 5B, D).

### LPS/CSE-induced cell death is associated with DAMP release

**Fig. 6:**
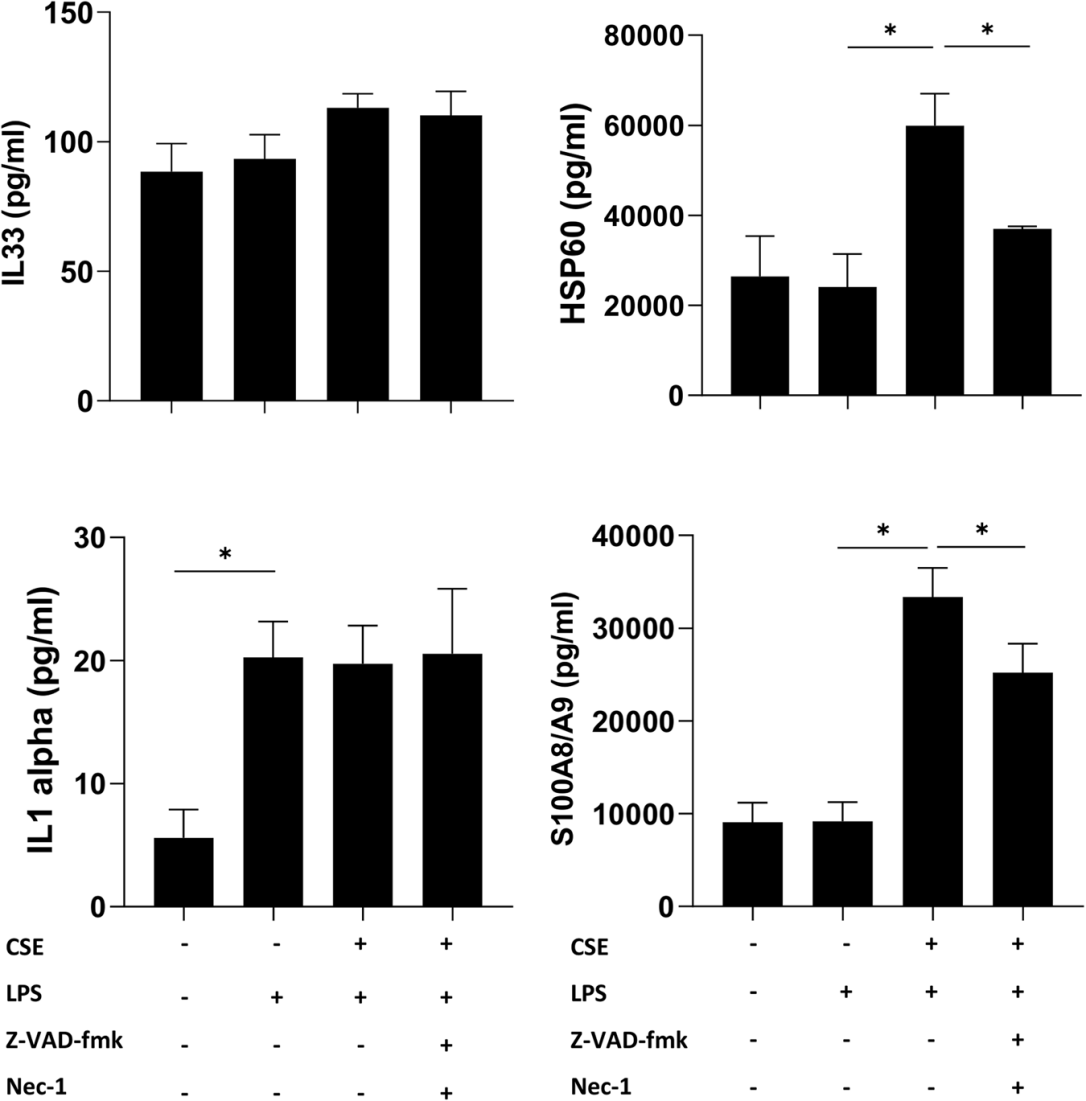
DAMPs are released from hMDMs exposed to LPS and CSE. Release of HSP60, S100A8/A9, IL1 alpha and IL33 in hMDMs exposed to 1 μg/ml of LPS and 20% CSE, alone or in combination for 24 h, evaluated by multiplex analysis. Where indicated, cells were pre-treated for 1 h with 20 μM Z-VAD-fmk pan-caspase inhibitor and 50 μM Nec-1 RIPK1 inhibitor. Data are presented as mean ± SEM (*N* = 3 independent donors).

Data so far reported indicated that CSE induced pro-inflammatory cell death after 24 h exposure to LPS, which is known to be associated to DAMP release [12]. Thus, the impact of cell death on the release of DAMPs as IL1 alpha, IL33, HSP60 and S100A8/A9 was evaluated in hMDMs treated with LPS and CSE, alone or in combination, and rescued or not with Z-VAD/Nec-1 (Fig. 6). The release of IL33 was not modulated in all tested conditions. The release of IL1 alpha was increased by LPS and not modulated by CSE. The release of HSP60 and S100A8/A9 only occurred after stimulation with LPS in combination with CSE and was significantly reverted upon rescue from cell death.

## Discussion

AMs are the most abundant immune cells in the lung under homeostatic conditions. As sentinel cells, they play an important role of gatekeeping and are the first line of defense against endogenous and exogenous threats including pathogens, necrotic cells/tissue, inhaled particles, and other environmental factors [1, 26, 28]. AMs’ ability to induce immune response relies on specific functions as phagocytosis and secretion of inflammatory mediators including reactive oxygen species (ROS) and pro-inflammatory cytokines (e.g., IL1 beta, IL18, IL6 and TNF), while efferocytosis and release of anti-inflammatory factors (e.g., IL10 and TGF beta) ensure a proper resolution of the inflammatory response [1, 25].

CS exerts a plethora of effects on AMs as changes in phenotype, activation profile, phagocytosis and efferocytosis ability, ROS production, imbalance of proteinase/anti-proteinase release, consequently affecting pathogen clearance and inflammation resolution [1, 8–11, 29, 30]. This predisposes smoker subjects to infections, often with very severe symptoms and high probability of chronicization. It is known that COPD patients go through periods of acute disease worsening, known as exacerbations, which follow bacterial/viral infection and increase morbidity and mortality [31]. Understanding molecular alterations underlying lung macrophage dysfunction induced by CS and responsible for the dysregulated inflammatory responses to pathogen-associated molecular patterns (PAMPs) may unveil novel pathogenic mechanisms and help developing more effective therapies.

Cell death physiologically regulates tissue homeostasis, response to infections and tissue repair after injury. However, if out of control, it contributes to propagate inflammation [12]. Many studies suggest that lytic forms of cell death may contribute to development and progression of chronic inflammatory diseases [12].

We had previously reported that CSE promoted inflammasome-independent activation of caspase-8, caspase-1 and GSDMD in hMDMs upon exposure to LPS via the TRIF pathway while inhibiting the TLR4-MyD88 axis [7, 19]. Herein, we tested the hypothesis that CSE may enhance pro-inflammatory cell death in response to LPS and this may contribute to propagate inflammation. To test this hypothesis, the impact of CSE on fate of human macrophages exposed to gram negative-derived LPS, was evaluated. Of note, treatment with LPS alone for 6 or 24 h did not induce cell death, nor caspase activation. On the contrary, in the presence of CSE, LPS induced lytic cell death associated with a strong activation of caspase-8 and -3/7 and morphological features of apoptosis. These data suggest that macrophages exposed to CSE are predisposed to the activation of cell death pathways upon encountering PAMPs. Caspase-8 is central in the regulation of several cell death pathways: it cleaves caspase-3/7 promoting apoptosis and activates GSDMD, either directly or indirectly via caspase-1, leading to pyroptosis, a highly inflammatory form of cell death [13]. In addition, depending on the expression of GSDME and the cell type, caspase-8 can promote the progression into pyroptosis inducing caspase-3-dependent cleavage of GSDME [32–34]. On the other hand, when activation of caspase-8 is inhibited, the phosphorylation cascade RIPK1/RIPK3/MLKL initiates, and necroptosis occurs as a backup death mechanism [13–15]. Our data showed that when CSE-exposed macrophages were treated with LPS, activation of caspase-8-dependent cell death occurred. We found that inhibition of caspase-8 with Z-IETD or Z-VAD pan-caspase inhibitor did not reduce cell death, hypothesizing a shift to necroptosis. Consistently, the addition of the RIPK1 inhibitor Nec-1 rescued cells from death thus confirming our hypothesis. Some reports have shown that under specific conditions RIPK1 may drive pyroptosis and apoptosis [35–37]. Our results show that LPS/CSE induced death and caspase-8 activation were partially inhibited by Nec-1. This suggests that RIPK1 is partially responsible for caspase-8 activation and cell death after LPS/CSE stimulation.

Among molecular mechanisms likely triggered by CSE that may enhance caspase-8 activation in response to LPS, cFLIP captured our attention. cFLIP is an anti-apoptotic factor and key interactor of caspase-8 [14] coded by *CFLAR*, whose expression is regulated by the TLR4/MyD88 pathway [17, 18, 38]. It has been reported that under conditions where the MyD88 pathway is inhibited, such as the treatment with the TAK1 inhibitor 5Z-7-Oxozeaenol (5z7), downregulation of CFLAR promotes caspase-8-dependent cell death in response to LPS in murine macrophages [39]. Inspired by this work, we hypothesized that CSE may contribute to caspase-8 activation by inhibiting LPS/MyD88-dependent induction of *CFLAR* gene. Results herein reported confirmed this hypothesis and showed that CSE completely blocked the induction of *CFLAR* thus revealing a mechanism through which CSE increased caspase-8 activity.

The observed activation of caspase-8 and 3/7 was accompanied by the presence of the major apoptosis hallmarks, including nuclear DNA condensation, fragmentation, and loss of MMP [22, 23, 40–42]. Of note, mitochondria membrane depolarization was also observed in hMDMs treated with CSE alone. These data, together with results of caspase-8 activation/cleavage, indicated that CSE alone caused mitochondrial damage, and this was associated with activation of caspase-8 in the absence of cell death. This may represent a sort of “priming” that causes CSE-sensitized cells [43–45] to rapidly activate cell death pathways upon encountering PAMPs as LPS.

Yet, the significant release of LDH in response to LPS/CSE indicated that apoptosis turned out into a lytic form of cell death associated with DAMP release. As mentioned before, caspase-8 is central in most cell death pathways [13, 15, 46]. We have previously reported that a short stimulation (6 h) with LPS/CSE led to sublytic activation of GSDMD, associated with increased cell permeability indicating the activation of a pyroptotic pathway [19]. At longer timepoints (24 h), exposure to LPS/CSE caused a potent caspase-8-dependent activation of caspase-3 suggesting a switch to apoptosis as confirmed by the observed morphological features. Activation of caspase-3 committed the cell to death and was associated with GSDME cleavage. To what extent the activation of GSDME downstream of apoptotic caspase-3 contributes to cell death in macrophages is still a matter of debate [37, 47]. Our data suggest that the cleavage of GSDME downstream of caspase-3 was not strictly required for the cell to undergo LPS/CSE-induced cell death.

Our findings are in line with previous reports. In particular, the cell death mechanisms that we observed in response to LPS/CSE appeared in some aspects similar to those observed in murine macrophages following stimulation with LPS combined with the TAK-1 inhibitor 5z7. In these conditions, cell death is characterized by a strong activation of caspase-8 (associated with CFLAR inhibition) leading to activation of caspase-3 and GSDME and cleavage of GSDMD [17, 36, 37]. Under these circumstances, despite being processed by caspase-3, GSDME appears to be dispensable for cell death as demonstrated using Gsdme^−/−^ macrophages [37]. Therefore, the exact role of GSDME cleavage downstream of caspase-3 in macrophages still remains elusive [37, 47–49].

A limitation of our study may be represented by the fact that the sensitivity of the LDH assay is not sufficient to detect small changes in cell death kinetics. Nevertheless, our results show that GSDME, although it may be a contributor, is not a primary driver of LPS/CSE- induced cell death. Importantly, one thing to be considered is that exposure to CSE alone significantly compromised the integrity of the mitochondrial membrane (Fig. 2D) therefore creating a condition that, combined with CFLAR inhibition, burst caspase-3 activation upon exposure to LPS, irreversibly committing cell to death. Under these circumstances, cell death cannot be rescued with single interventions such as the silencing of GSDME. In this respect it has been widely demonstrated that the extensive crosstalk among cell death pathways allows the cell to rapidly switch from a type of death to another when a death pathway is blocked by pharmacological intervention or gene knocking down [15, 50–54]. This is most likely what is occurring in LPS/CSE stimulated cell death, which can be rescued only by the combined blockade of all caspases as well as the necroptotic pathways. Overall, our data suggest that pyroptosis may be initially contributing to cell death in human macrophages exposed to CSE/ LPS while leaving the stage to caspase-8-dependent apoptosis and secondary necrosis at later timepoints.

Notably, cleaved caspase-3 was found increased in alveolar macrophages of a small group of lung tissue sections from smoking subjects compared to non-smoker controls. We also investigated how cigarette smoking impacts alveolar macrophage transcriptional profile by interrogating two publicly available datasets, GSE13896 [11] and GSE2125 [20]. The analysis of differentially expressed genes of merged datasets revealed that *GSDME* was significantly increased in Smokers compared to Non-Smokers and the GO analysis revealed the involvement of differentially expressed genes in cell death processes. Taken together, these findings show that AMs of smokers display features indicative of cell death dysregulation.

Many studies demonstrated that lung macrophages from smokers display defective acute inflammatory response to LPS [11]. Herein, we confirmed that CSE strongly inhibited the early inflammatory response to LPS. Interestingly, we found that the release of pro-inflammatory cytokines such as IL6 and TNF raised up at longer exposure times. This may derive from the residual gene expression leading to the slow accumulation of cytokines in the extracellular space, reflecting an impaired inflammatory response. Pro-inflammatory cell death is known to be associated with DAMP release [12], and high levels of DAMPs including S100 proteins, defensins, and high-mobility group box-1 (HMGB1) were found in extracellular lung fluids of patients with chronic inflammatory lung diseases as COPD [54]. Herein we show increased release of S100A8/A9 and HSP60 after stimulation with LPS in presence of CSE but not when cells were exposed to CSE or LPS alone. Release of S100A8/A9 and HSP60 was reverted after cell rescue by pre-treatment with Z-VAD and Nec-1, suggesting a strict correlation with cell death. It has been recently demonstrated that GSDMD pores play a key role on secretion of the complex S100A8/A9 [55]. GSDMs pore formation may contribute to mediate S100A8/A9 secretion in hMDMs exposed to CSE and stimulated with LPS. Future investigations may help highlighting the potential role of GSDMs on S100A8/A9 release from hMDMs in cigarette smoke-associated inflammatory responses.

Overall, our data suggest that in addition to significantly impairing the early inflammatory response to PAMPs, CSE sensitizes human macrophages to caspase-8-dependent lytic cell death leading to DAMP release in response to bacterial LPS. Translating these findings into the context of airways infections in smokers suggest that while a delayed inflammatory response may result in ineffective defenses toward infections, the occurrence of pro-inflammatory cell death may contribute to perpetuate a state of unresolved and chronic inflammation. Future investigations on the impact of CSE-induced pro-inflammatory cell death on surrounding cells will provide new insights on the molecular mechanisms promoting CS-associated lung chronic diseases. Fine tuning macrophage cell death may represent a new potential way to improve response to infection and control chronic inflammatory diseases.

## Materials and methods

### Reagents and antibodies

RPMI 1640 medium (ECB9006L), L-glutamine (ECB3000D), penicillin–streptomycin (ECB3001D), fetal bovine serum (FBS) (ECS5000L), sodium pyruvate (ECM0542D), HEPES (ECM0180D), and Dulbecco’s Phosphate Buffer Saline w/o Calcium w/o Magnesium (ECB4004L) were purchased from Euroclone (Milan, Italy). Human M-CSF (130-096-493) was purchased from Miltenyi Biotec (Bergisch Gladbach, Germany). The following chemicals were obtained from Sigma-Aldrich (Missouri, USA): lipopolysaccharides from Escherichia coli 0111:B4 (LPS, L3012), Z-VAD-fmk Caspase Inhibitor I (627610), Z-IETD-fmk Caspase-8 Inhibitor II (218759), Necrostatin-1 (Nec-1) (N9037), and Actinomycin D (ActD) (A1410). Primary antibodies for western blot were as follows: antibody against caspase-8 (ALX-804-242-C100) was from Enzo Life Sciences (Euroclone, Milan, Italy), antibody against cleaved caspase-3 (9661S) was from Cell Signaling (Euroclone, Milan, Italy), antibody against GSDME (ab215191) was from Abcam (Prodotti Gianni, Milan, Italy), and actin (sc-81178) from Santa Cruz Biotechnology (Dallas, TX, USA). Primary antibodies were used at the following dilutions: anti-actin 1:10,000, anti-caspase-8 1:100, anti-cleaved caspase-3 1:500, anti-GSDME 1:1000. The following secondary antibodies for western blot assay were purchased from LI-COR (Lincoln, Nebraska, USA): Goat Anti-Mouse IRDye 680RD (926-68070) and Donkey Anti-Rabbit IRDye 800CW (926-32213). Secondary antibodies were used at the following dilutions: 1:2000 for anti-mouse and 1:5000 for anti-rabbit. Hoechst fluorescent nucleic acid stain (33342) was obtained from ImmunoChemistry technology, Davis, California, USA. JC-1 Dye (Mitochondrial Membrane Potential Probe) (T3168) was purchased from Invitrogen (Thermo Fisher Scientific, Waltham, Massachusetts, USA).

### Human monocyte-derived macrophages (hMDMs)

Peripheral blood mononuclear cells (PBMCs) were isolated from buffy coats derived from three healthy donors and received by ARNAS “Civico, Di Cristina, Benfratelli” (Palermo, Italy) according to a Material Transfer Agreement signed on 8/11/2019.

Human macrophages were obtained by culturing PBMCs for 7 days in complete RPMI 1640 medium supplemented with 10% FBS and 50 ng/ml of human M-CSF. The medium was replaced after 3 days of culture. The day before each experiment, hMDMs were treated with trypsin-EDTA for 5 min, scraped, plated in complete medium without M-CSF into 96-well plates (5 × 10^4^ cells/well) or 6-well plates (1.5 × 10^6^ cells/well), and incubated at 37 °C and 5% CO_2_. The day of the experiment, culture medium was changed to 1% FBS and cells were stimulated for 24 h, unless otherwise indicated for 6 and 24 h, as follows: 1 μg/ml LPS, in presence or not of 20% CSE, which was obtained as previously described [7]. Where indicated, 0.1 μM of Z-IETD-fmk Caspase-8 Inhibitor II, 20 μM of Z-VAD-fmk Caspase Inhibitor I, in combination or not with 50 μM of RIPK1 inhibitor Nec-1, were added 1 h before LPS/CSE stimulation. Where indicated, 0.5 μg/ml of ActD was used as positive control and added concomitantly with LPS/CSE stimulation.

### Lung tissue samples

Lung tissue samples were obtained from patients undergoing surgery for lung cancer. Sampling did not interfere with the subsequent examinations of the specimens by the pathologist. The study protocol was approved by the Institutional Review Board for human studies at IRCCS ISMETT (IRRB/19/19) and conducted in accordance with the Declaration of Helsinki. Informed written consent was obtained from each patient. The patients were grouped as follows: never smoking patients (Non-Smokers, *N* = 6); smoker patients (>15 pack/year) (Smokers, *N* = 5). Characteristics of patients are reported in Table S1. The presence of COPD patients in selected groups was excluded by performing spirometry evaluation. COPD patients were classified based on GOLD Guidelines 2019 (https://goldcopd.org/pocketguidereferences/gold-2019-pocket-guide-references/): Forced expiratory volume in one second (FEV_1_) less than 80% of reference, FEV_1_/Forced Vital capacity (FVC) less than 70%, and bronchodilatation effect less than 12%. All recruited subjects had negative skin tests for common aeroallergen extracts and had no history of asthma and/or allergic rhinitis. The patients were not under corticosteroid therapy (inhaled or systemic) and were not under antibiotics. Patients did not have exacerbations during the month preceding the study.

### Isolation of total RNA and real-time quantitative PCR (RT-qPCR)

Total RNA was extracted using TRIzol Reagent (Invitrogen) following the manufacturers’ instruction. One μg of total RNA was reverse transcribed to cDNA in a volume of 20 μl, using the iScript cDNA Synthesis kit (Bio-Rad, Hercules, California, USA). Quantitative PCR was carried out on Step One Plus Real-time PCR System (Applied Biosystems, Foster City, California, USA) using specific FAM-labeled probe and primers: pre-validated TaqMan Gene expression assay was used for *CFLAR* (Hs01116280_m1), *TNF* (Hs99999043_m1) and *IL6* (Hs00985639_m1) (Applied Biosystems). Gene expression was normalized to GAPDH: pre-validated TaqMan Gene expression assay was used for *GAPDH* (Hs03929097_g1) (Applied Biosystems) endogenous control gene. The expression of target genes was calculated using the ΔCt method.

### Analysis of secreted proteins

ELISA assay was performed for the detection of TNF, IL6 and S100A8/A9 following the manufacturers’ instructions (R&D Systems, Minneapolis, Canada).

The levels of different secreted soluble factors were determined in hMDM supernatants by multiplex analysis. Luminex™ magnetic bead technology combined with a ProcartaPlex™ Multiplex Immunoassay (Invitrogen) was used according to the manufacturer’s instructions. Briefly, each analyte was quantified using a Luminex 200 instrument, which utilizes xMAP technology, multiple analyte profiling and xPONENT 4.2 software (Luminex Corp., Austin, Texas, USA). Concentration of each protein was calculated using standard curves.

### LDH and caspase activity assays

LDH released from cells was measured using the CytoTox 96® Non-Radioactive Cytotoxicity Assay LDH Cytotoxicity Assay Kit (G1780, Promega) according to the manufacturer’s protocol. The absorbance of LDH was measured at 490 nm.

The extracellular activity of caspase-8 and 3/7 was determined using respectively Caspase Glo 8 and 3/7 homogeneous luminescent assay kits (Cat# G8202, Cat# G8093, Promega Corporation, Madison, Wisconsin, USA), following the manufacturers’ instruction [56].

### Immunohistochemistry

For immunohistochemistry (IHC), 5-µm formalin-fixed and paraffin-embedded peripheral lung sections were deparaffinized in xylenes and rehydrated through a series of graded alcohol. The sections were initially treated at 75 °C in sodium citrate (pH 6.5) for antigen retrieval. After being washed, the sections were incubated with the primary antibody specific for cleaved Caspase-3 (9661S Cell Signaling Technologies, 1:500) at 4 °C overnight. Immunoreactivity was visualized using a Mouse and Rabbit Specific HRP/DAB Detection IHC kit (ab64264, Abcam) according to the manufacturer’s instructions and counterstained with Mayer’s Hematoxylin for 45 s. Finally, the slides were prepared for observation with coverslips, using a permanent mounting medium (Vecta Mount, Vector, H-5000).

The sections were observed, and pictures were taken using digital EVOS M5000 imaging system (Thermo Fisher, AMF5000). Three independent observers (A.L.M., M.R.G. and F.R.) evaluated the reactions and performed an immunomorphological evaluation to distinguish the cleaved caspase-3-positive cells. A quantitative analysis to determine the percentage of immunopositivity was also carried out. All evaluations were made at a magnification of ×40. The arithmetic mean of counts was used for statistical analysis. The statistical difference between the percentage of immunopositivity in non-smoker controls and smokers was evaluated with the Mann–Whitney test using GraphPad Prism 9.0.1 (GraphPad Software Inc., San Diego, California).

### Western blot

Cells were lysed using a lysis buffer made as follows: 10 mM Tris–HCl, 50 mM NaCl, 5 mM EDTA, 1% Nonidet P-40, protease inhibitor (P8340, Sigma-Aldrich), and phosphatase inhibitor (P0044, Sigma-Aldrich). Total protein content was determined using the Bradford protein assay kit (Thermo Fisher Scientific). A total of 30 μg protein was loaded, resolved by SDS-PAGE, and blotted onto nitrocellulose membranes (Bio-Rad). For supernatant precipitation, cells were stimulated in the medium with 1% FBS, and samples were processed as previously described [57]. Blots were incubated overnight with primary antibodies at 4 °C. The blots were analyzed using the Odyssey Imaging System (LI-COR). Full and uncropped western blots are presented in Supplemental File Uncropped WB.

### Analysis of nuclear morphology

The assay was performed in 96-well plates (PhenoPlate 96-well, black, optically clear flat-bottom, PerkinElmer, Waltham, Massachusetts, USA). After stimulation, cells were stained with Hoechst 33342 1.5 μg/ml. Nuclear DNA condensation was measured by Harmony Software (Perkin Elmer) as mean fluorescence intensity (MFI) of the nucleus stain. Image acquisition was performed using Operetta CLS (Perkin Elmer) with a 63X objective.

### Mitochondrial membrane potential (MMP)

The assay was performed in 96-well plates (PhenoPlate 96-well, black, optically clear flat-bottom, Perkin Elmer). After stimulation, cells were incubated with 10 μM of JC1 dye + 1.5 μg/ml of Hoechst 33342 in PBS for 15’ at 37 °C, and then gently washed with PBS. Mitochondrial membrane potential, indicated by the red (485/590 nm)/green (485/529 nm) fluorescence intensity ratio was measured by TECAN plate reader. Live images were acquired using Operetta CLS (Perkin Elmer) using a 63X objective.

### Statistical analysis

Statistical analysis was performed using GraphPad Prism 9.0.1 (GraphPad Software Inc., San Diego, California). For each of the three independent donors, *N* = 3 technical replicates were performed. Regarding sample size of human lung tissues, all available samples provided by the hospital were used. Unless otherwise stated, data were expressed as mean ± SEM. Differences were identified using one‐way repeated measures ANOVA and Tukey’s post hoc multiple comparisons test. The equality of group variances has been tested with Brown-Forsythe test. Differences with *p* < 0.05 were considered significant. Preprocessing and analysis of publicly available microarray datasets is described in Supplementary Information.

### Supplementary information

Supplemental table

Supplemental data and material

Supplemental file Uncropped WB

Author Checklist
