# Supplementary material for: Caspase-8 activation by cigarette smoke induces pro-inflammatory cell death of human macrophages exposed to lipopolysaccharide
Source: Cell Death Dis. 2023 Nov 25;14(11):773. doi: 10.1038/s41419-023-06318-6 (PMC10676397; doi:10.1038/s41419-023-06318-6)
Supplement: Supplementary file 1 — Supplemental table [file 41419_2023_6318_MOESM1_ESM.docx]

**Supplementary table**

| **Table S1.** Characteristics of patients | | |  |  |  |  |
| --- | --- | --- | --- | --- | --- | --- |
|  | **Age** | **Gender (M/F)** | **Pack/years** | **FEV1 L** | **FEV1 % pred** | **FEV1/FVC %** |
| Non-Smokers (6) | 62 ± 8.8 | 1/5 | - | 2.4 ± 0.7 | 116 ± 15.6 | 106.6 ± 13.2 |
| Smokers (5) | 63 ± 4.5 | 2/3 | 31 ± 15.6 | 2.4 ± 0.5 | 107.2± 9.7 | 89.1 ± 10.9 |
