# Supplementary material for: Caspase-8 activation by cigarette smoke induces pro-inflammatory cell death of human macrophages exposed to lipopolysaccharide
Source: Cell Death Dis. 2023 Nov 25;14(11):773. doi: 10.1038/s41419-023-06318-6 (PMC10676397; doi:10.1038/s41419-023-06318-6)
Supplement: Supplementary file 2 — Supplemental data and material [file 41419_2023_6318_MOESM2_ESM.docx]

**Supplementary data**


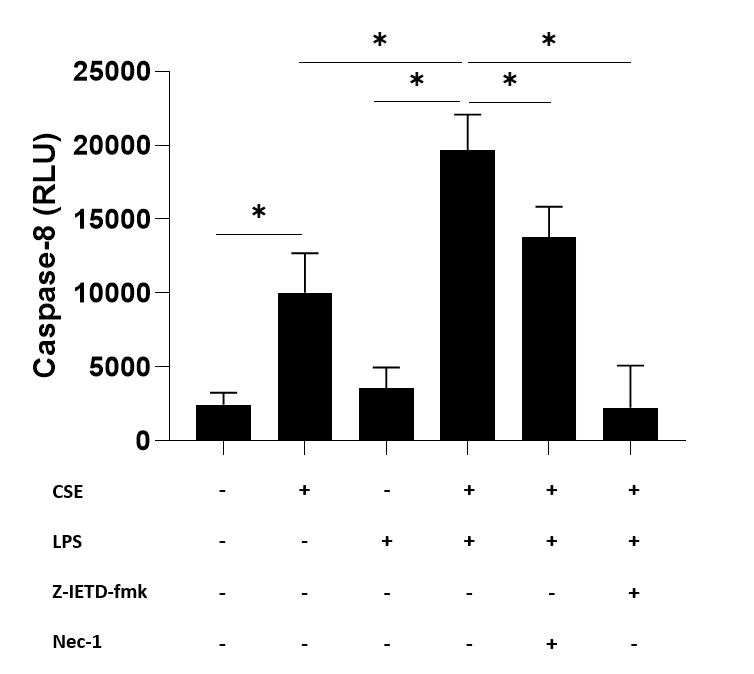


**Figure S1 Nec-1 reduces caspase-8 activation in LPS/CSE-treated hMDMs.** Extracellular activity of Caspase-8 (expressed as relative luminescence unit, RLU), in hMDMs treated with 1 μg/mL of LPS and 20% CSE, alone or in combination for 24h. Where indicated, cells were pre-treated for 1 hour with 0.1 μM Z-IETD-fmk Caspase-8 inhibitor or 50 μM Nec-1 RIPK1 inhibitor. Data are presented as mean ±SEM (N= 3 independent donors).


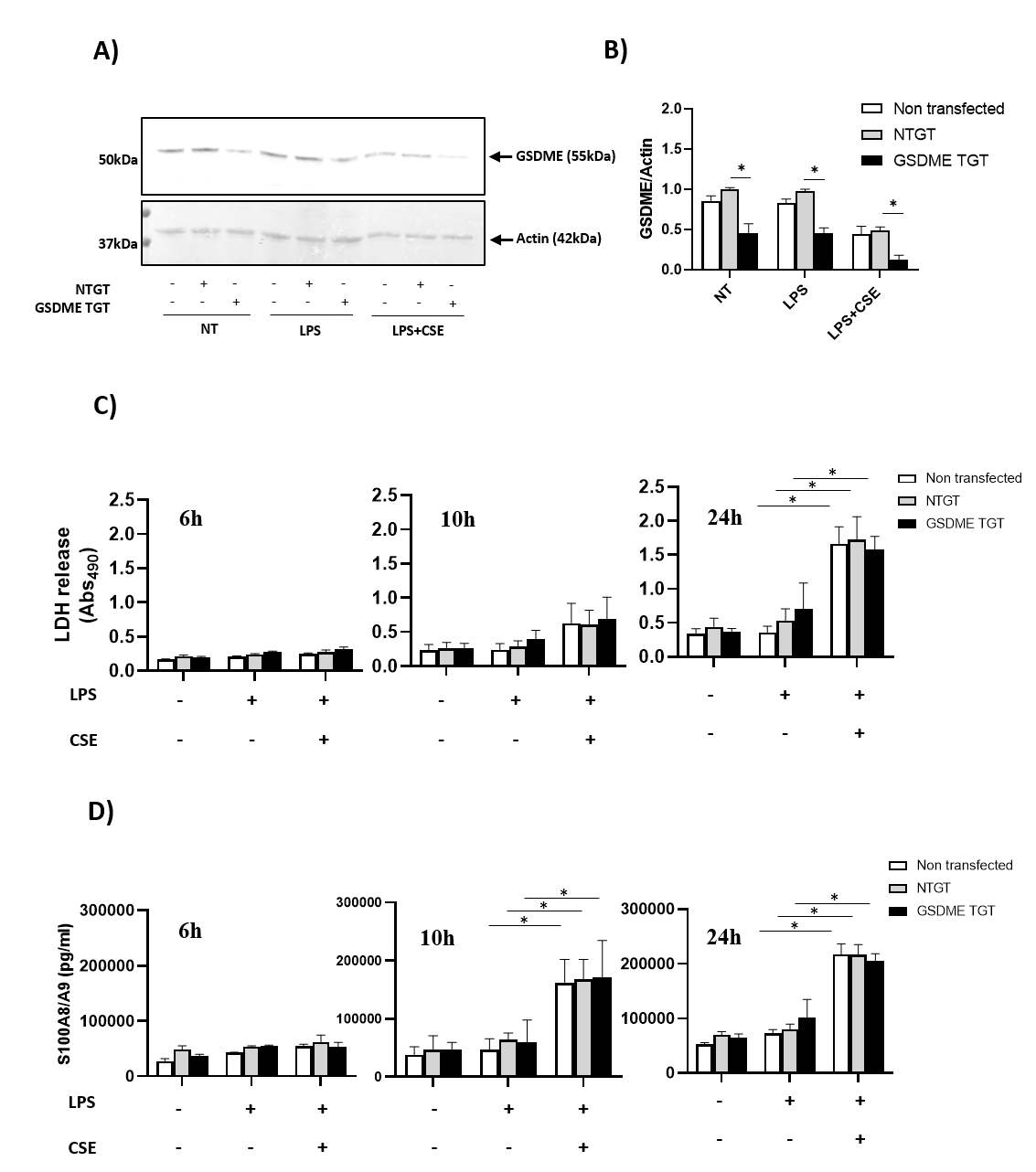


**Figure S2 Impact of GSDME silencing on LPS/CSE-induced cell death.** hMDMs were transfected with 350 nM of Gasdermin E targeting siRNA (GSDME TGT) or Non-Targeting (NTGT) control and, where indicated, stimulated with 1 μg/mL of LPS, alone or in combination with 20% CSE for 24h. **A)** Representative western blot images and **B)** densitometric analysis of GSDME in silenced hMDMs (N=3 independent donors); **C)** LDH and **D)** S100A8/A9 release at 6h, 10h and 24h after treatment with LPS, alone or in combination with 20% CSE. Data are presented as mean ±SEM (N= 3 independent donors).


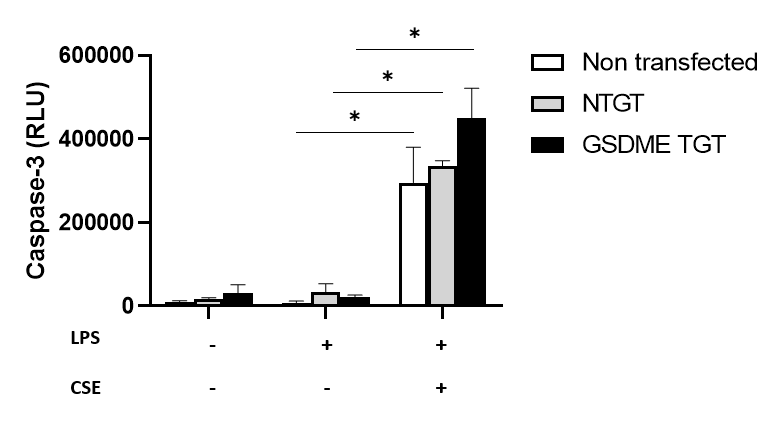


**Figure S3 Impact of GSDME silencing on LPS/CSE-induced activation of caspase-3.** Extracellular activity of Caspase-3/7 (expressed as relative luminescence unit, RLU) in hMDMs transfected with 350nM of GSDME TGT or NTGT siRNA and stimulated with 1 μg/mL of LPS, alone or in combination with 20% CSE for 24h. Data are presented as mean ±SEM (N= 3 independent donors).


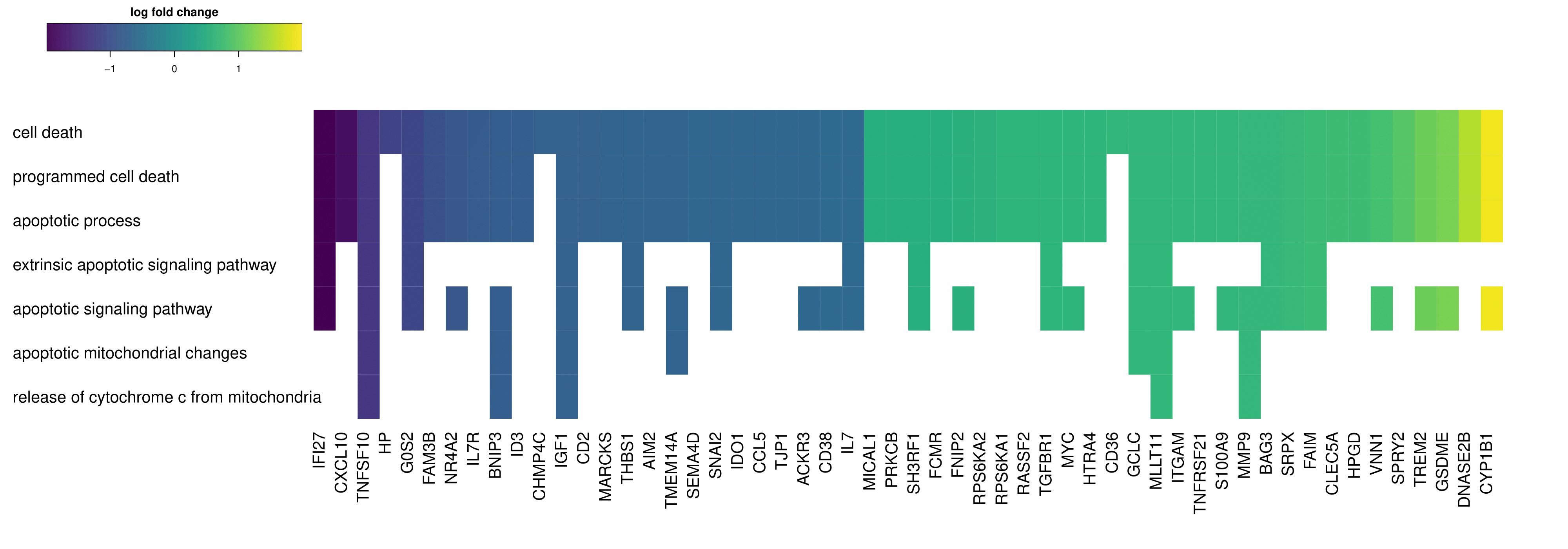


**Figure S4 Gene ontology enrichment analysis results**. The heatmap shows the GO terms related to cell death term (GO:0008219). Genes are sorted by log fold change values computed by Smokers vs Non-Smokers gene expression comparison. The cells in the heatmap are colored if the correspondent gene belongs to the correspondent GO term, are white if the gene doesn’t belong to the GO term.

**Supplementary material**

**hMDM small interfering RNA transfection**

1.5 x 10^6^ cells/well were seeded in 6-well plates. hMDMs were transfected with 350 nM of Gasdermin E targeting siRNA (GSDME TGT) (FE5L011844000005 Dharmacon Horizon) and Non-Targeting (NTGT) control (FE5D0018101020 Dharmacon Horizon), following the manufacturer’s instructions. Lipofectamine 3000 (L3000-008) from Life Technologies (Thermo Fisher Scientific) was used as transfection reagent. After 24h medium was removed and replaced with RPMI medium 10% FBS, without penicillin/streptomycin. Then, after 24h, the cells were stimulated with 1 μg/mL of LPS, alone or in combination with 20% CSE. LDH and S100A8/A9 release were measured in cell supernatants at 6h, 10h and 24h after treatment. Caspase 3/7 enzymatic activitywas measured in cell supernatants 24h after treatment.

**Bioinformatic methods**

**Datasets**

The raw data (.cel files) of two gene expression datasets were downloaded from the Gene Expression Omnibus (GEO) repository using the “GEOquery” R package with the following accession numbers: GSE13896 [1] and GSE2125 [2].

GSE13896 is a collection of transcriptional profiling of alveolar macrophages obtained by bronchoalveolar lavage of 24 healthy non-smokers, 34 healthy smokers, and 12 chronic obstructive pulmonary disease (COPD) smokers. The data from COPD samples was not used in this study.

GSE2125 collects the transcriptional profiling of alveolar macrophages of 15 healthy non-smokers, 15 healthy smokers, and 15 subjects with asthma. The data from asthmatic samples was not used in this study.

Both datasets were collected using the Affymetrix Human Genome U133 Plus 2.0 array (GPL570).

**Data pre-processing**

The raw data were pre-processed through the robust multichip average (RMA) pre-processing method from R-package “affy” [3] to perform background correction and normalization [4]. Non-annotated probes were excluded, whereas probes mapping the same gene were averaged.

Since a qualitative check of a low dimensional plot highlighted the existence of 2 clusters of samples driven by the two-accession number, data were adjusted for batch effect using the ComBat method [5].

**Differentially expressed genes analysis**

The “limma” R package [6] was used to identify the Differentially Expressed Genes (DEG) between smokers and non-smokers. The signiﬁcantly DEGs were identiﬁed by p-value FDR adjusted < 0.05 and |log2 fold change (FC)| > 0.5.

**Gene ontology enrichment analysis**

To investigate the biological meaning of the 304 DEGs found, a gene ontology (GO) enrichment analysis was performed by the enrichGO function in “clusterProfiler” package [7]. The research was limited to GO terms in Biological Process and returned terms with p-value FDR corrected < 0.05. From the file with all the significant terms, those related to the cell death term (GO:0008219) were filtered (Figure S1).

**Bibliography**

1. Shaykhiev R, Krause A, Salit J, Strulovici-Barel Y, Harvey B-G, O’Connor TP, Crystal RG (2009) Smoking-dependent reprogramming of alveolar macrophage polarization: implication for pathogenesis of chronic obstructive pulmonary disease. J Immunol 183:2867–2883

2. Woodruff PG, Koth LL, Yang YH, Rodriguez MW, Favoreto S, Dolganov GM, Paquet AC, Erle DJ (2005) A distinctive alveolar macrophage activation state induced by cigarette smoking. Am J Respir Crit Care Med 172:1383–1392

3. Gautier L, Cope L, Bolstad BM, Irizarry RA (2004) affy—analysis of Affymetrix GeneChip data at the probe level. Bioinformatics 20:307–315

4. Irizarry RA, Hobbs B, Collin F, Beazer-Barclay YD, Antonellis KJ, Scherf U, Speed TP (2003) Exploration, normalization, and summaries of high density oligonucleotide array probe level data. Biostatistics 4:249–264

5. Johnson WE, Li C, Rabinovic A (2007) Adjusting batch effects in microarray expression data using empirical Bayes methods. Biostatistics 8:118–127

6. Ritchie ME, Phipson B, Wu D, Hu Y, Law CW, Shi W, Smyth GK (2015) limma powers differential expression analyses for RNA-sequencing and microarray studies. Nucleic Acids Res 43:e47

7. Wu T, Hu E, Xu S, et al (2021) clusterProfiler 4.0: A universal enrichment tool for interpreting omics data. The Innovation 2:100141
