# Supplementary material for: Caspase-8 activation by cigarette smoke induces pro-inflammatory cell death of human macrophages exposed to lipopolysaccharide
Source: Cell Death Dis. 2023 Nov 25;14(11):773. doi: 10.1038/s41419-023-06318-6 (PMC10676397; doi:10.1038/s41419-023-06318-6)

Pro-caspase-8 (57kDa)

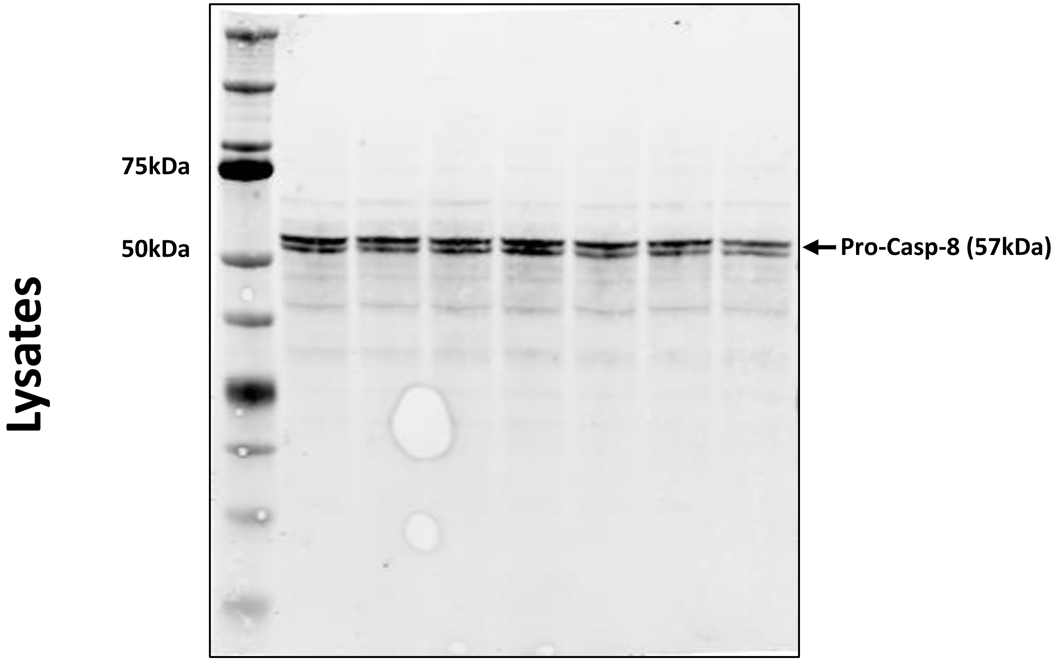

Cleaved caspase-3 (17-19kDa)

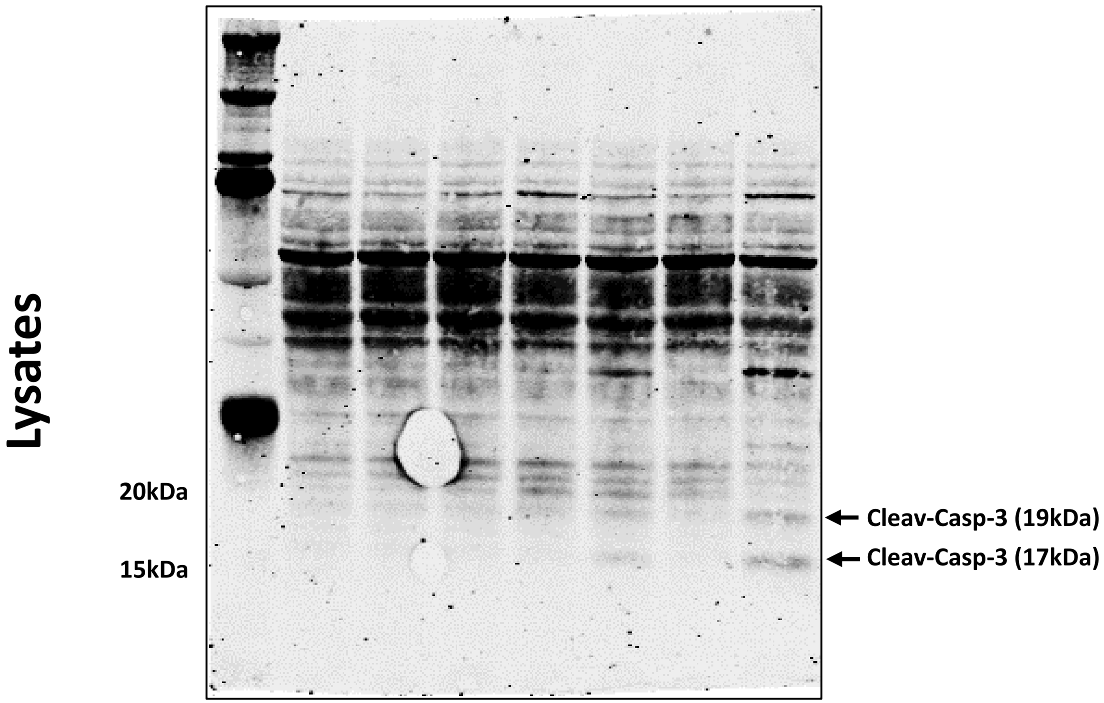

Full length GSDME (55kDa)
Cleaved GSDME (30kDa)

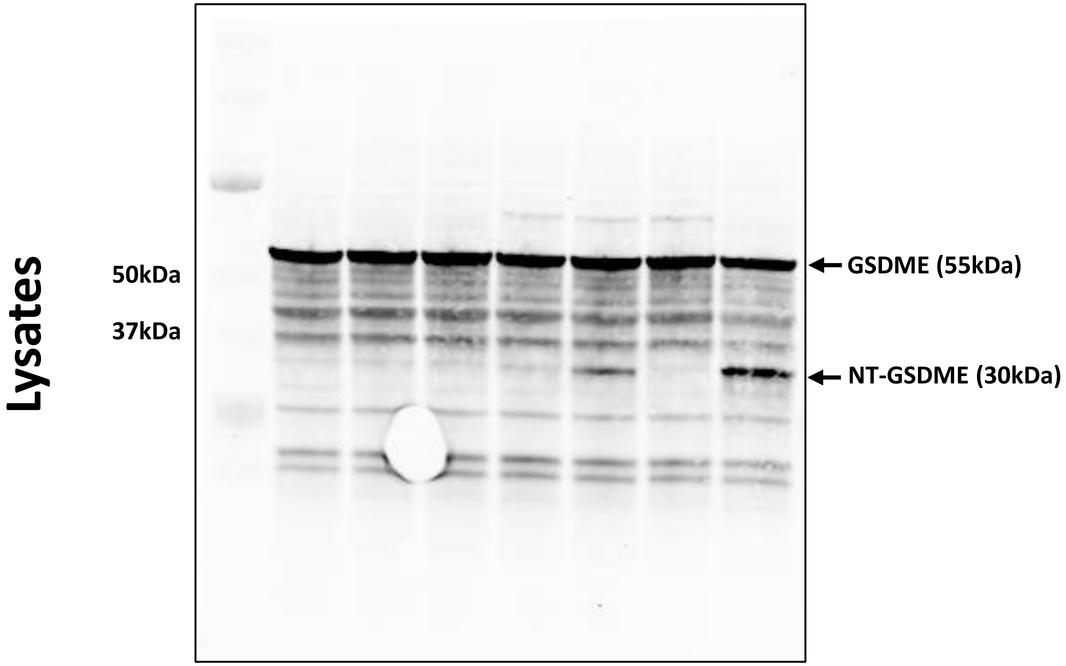

β-Actin (42kDa)

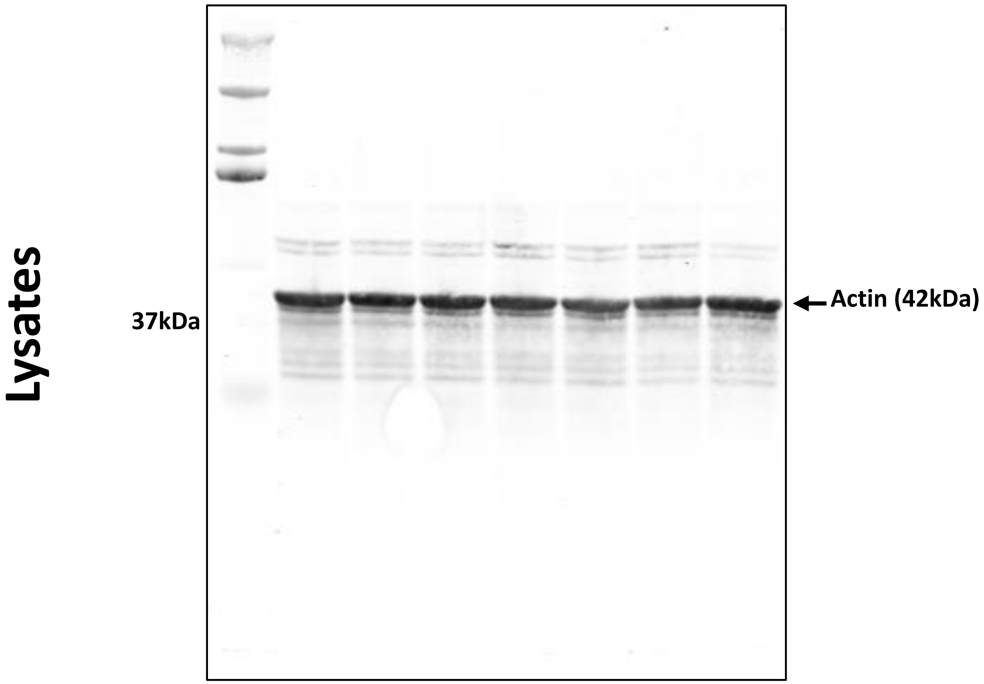

Cleaved caspase-8 (43-18kDa)

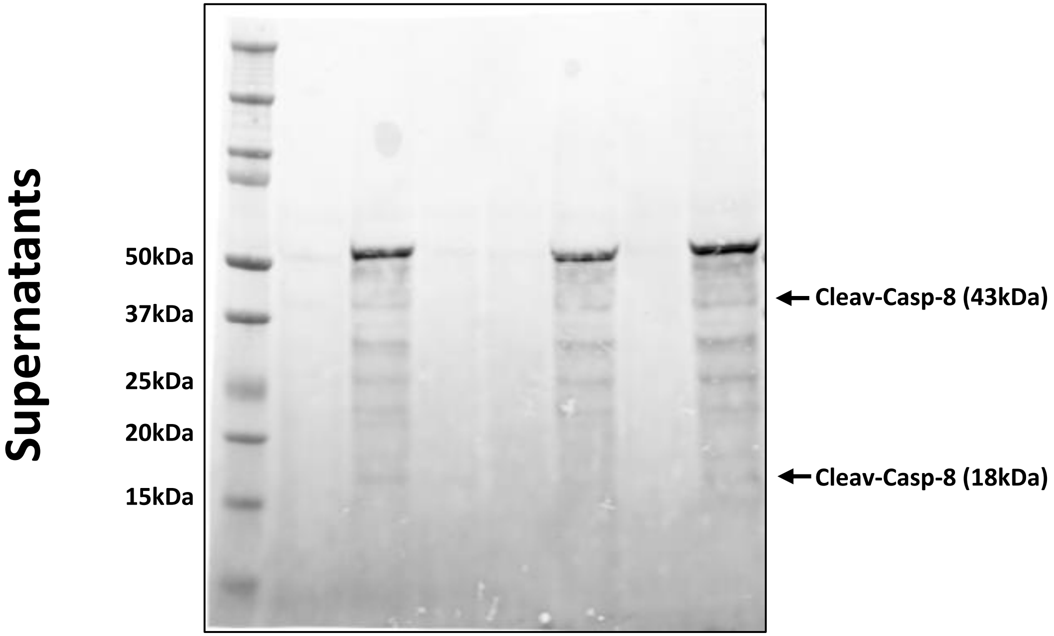

Full length GSDME (55kDa)

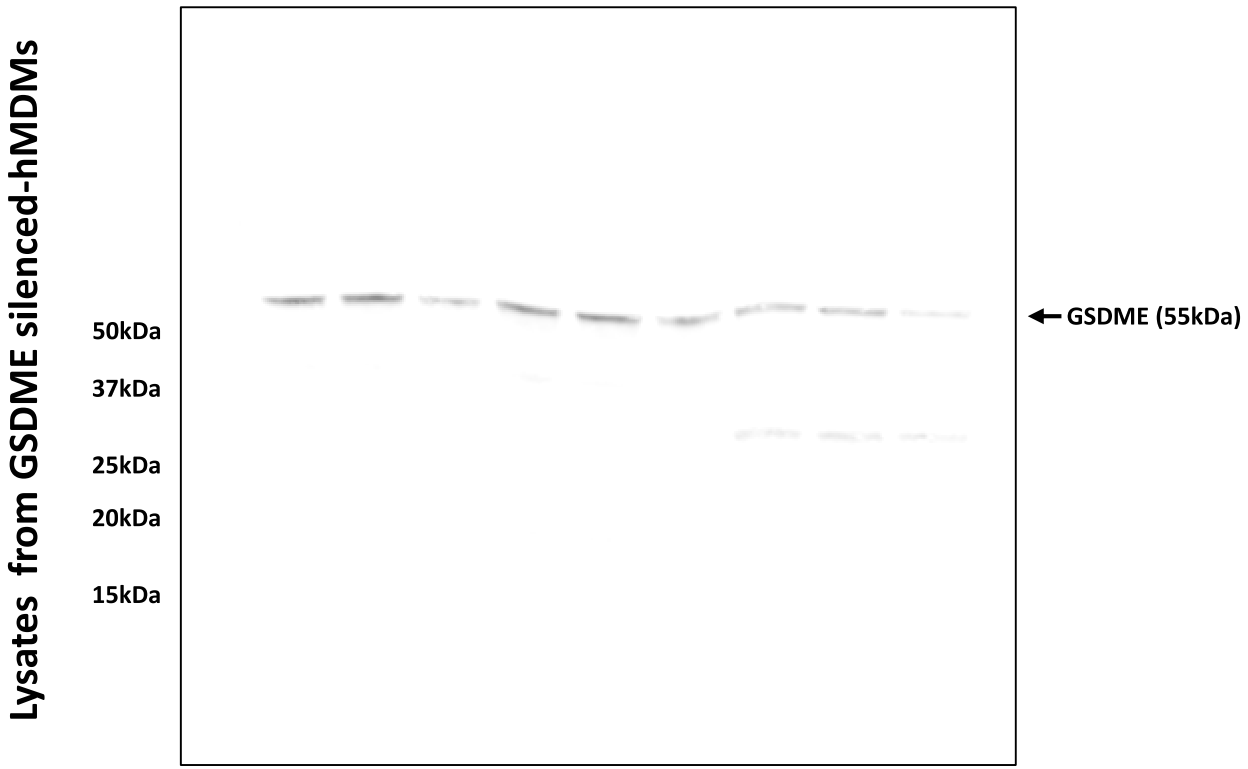

β-Actin (42kDa)

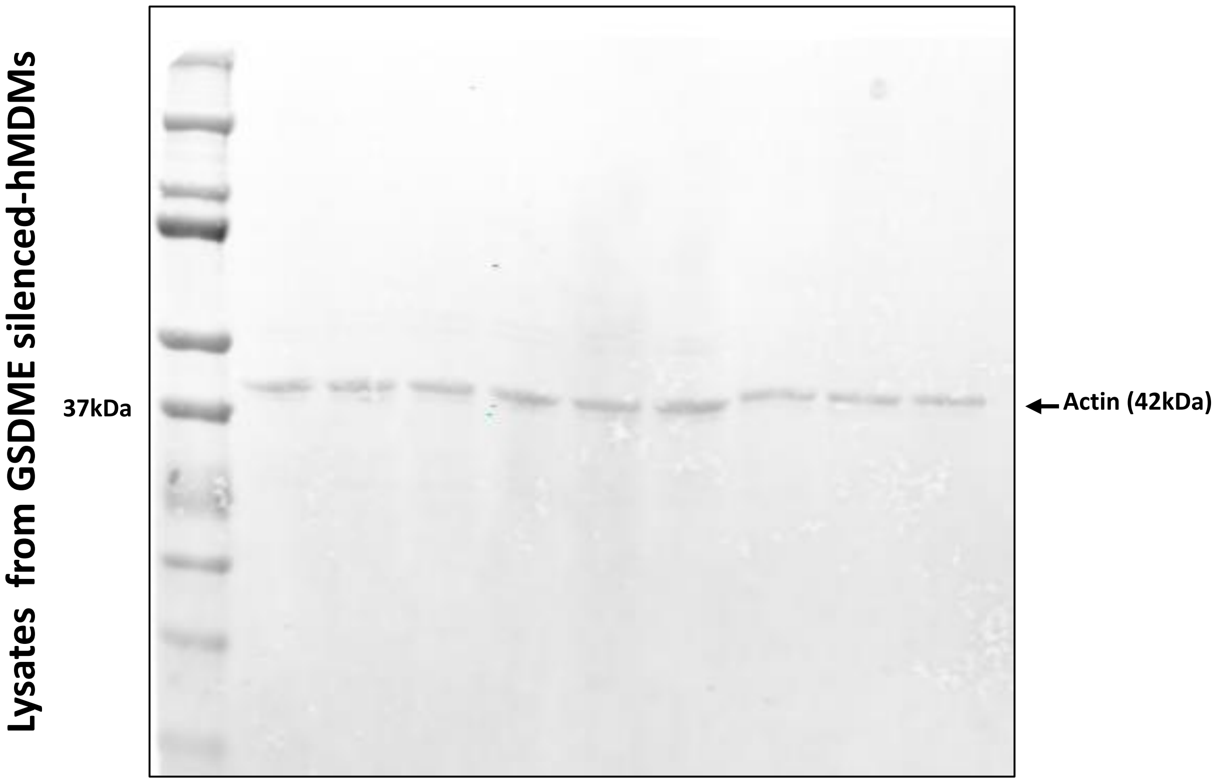

Supplement: Supplementary file 3 — Supplemental file Uncropped WB [file 41419_2023_6318_MOESM3_ESM.pdf]
